# Genetic diversity of norovirus in Shenzhen Based on continuous surveillance from 2016 to 2022

**DOI:** 10.3389/fcimb.2025.1593610

**Published:** 2025-06-18

**Authors:** Xin Wang, Wanqiu Liu, Mingda Hu, Hui Ma, Yaqing He, Boqian Wang, Kexin Li, Rui Zhang, JingJing Fu, Hailong Zhang, Long Chen, Xinru Zhao, Buaijier Aimaiti, Hongbin Song, Hongguang Ren, Xiaofeng Hu

**Affiliations:** ^1^ Laboratory of Advanced Biotechnology, Beijing Institute of Biotechnology, Beijing, China; ^2^ School of Public Health, University of South China, Hengyang, China; ^3^ Institute of Pathogen, Shenzhen Center for Disease Control and Prevention, Shenzhen, China; ^4^ Nursing Department, PLA General Hospital, Beijing, China; ^5^ Health Supervision and Surveillance, Chinese People's Liberation Army (PLA) Center for Disease Control and Prevention, Beijing, China; ^6^ School of Public Health, China Medical University, Shenyang, China; ^7^ School of Public Health,Southern Medical University, Guangzhou, China

**Keywords:** norovirus, genetic diversity, mutation, recombination, Shenzhen

## Abstract

**Introduction:**

Norovirus is a key pathogen of acute gastroenteritis and poses a significant burden on both the economy and public health. This study focuses on continuous monitoring of norovirus in Shenzhen, China, from 2016 to 2022, aiming to analyze the epidemic characteristics and genetic diversity of norovirus in the context of global sequence data.

**Methods:**

The study was based on data collected from local sentinel hospitals. It involved analyzing the demographic, spatial, and temporal distribution of norovirus infections. Phylogenetic analysis was conducted, and genotype dynamics were compared across geographic levels. Mutations affecting protein stability were evaluated, and recombination analysis was performed to identify critical breakpoints and fragments for norovirus.

**Results:**

The study found that norovirus primarily infected infants under 3 years old, with epidemics occurring in winter and concentrated in developed districts. Phylogenetic analysis revealed both similarities and differences in the evolutionary patterns of various genotypes at different geographical levels. Mutations in the VP1 protein, based on the protein structure of GII.4_Sydney[P31], provided insights into the evolutionary trends of key genotypes. Additionally, recombination analysis identified important breakpoints and fragments for norovirus.

**Discussion:**

The findings offer valuable insights to evolution and transmission of norovirus. These results can serve as a reference for future research, and they may aid in vaccine development efforts aimed at controlling norovirus outbreaks.

## 1 Introduction

Acute gastroenteritis (AGE) is the leading cause of illness and death among children under 5 years old, posing a heavy burden on the economy and public health (Liu et al., 2015). While gastroenteritis is typically mild and self-limiting, it can also cause hospitalization and death, particularly among infants and the elderly. With the gradual promotion of rotavirus vaccines, the detrimental effects of rotavirus have been significantly reduced. Consequently, human noroviruses (HuNoV) are recognized as the primary causative agent of acute gastroenteritis (Ahmed et al., 2014). It has been estimated that HuNoV is responsible for over 600 million AGE cases and approximately 21,000 deaths annually (Pires et al., 2015). Therefore, it is of vital importance to investigate the evolutionary characteristics of HuNoVs by continuous surveillance.

Noroviruses belong to the *Caliciviridae* family and are characterized as icosahedral, non-enveloped, single-stranded RNA viruses. The genome of HuNoV is typically 7.5–7.7 kb in length and consists of three open reading frames (ORF1–3). ORF1 encodes a polyprotein that includes NTPase, 3C-like protein, and RNA-dependent RNA polymerase (RdRp). ORF2 and ORF3 encode the major and minor capsid proteins, VP1 and VP2, respectively (Campillay-Véliz et al., 2020). Specifically, VP1 plays a significant role in viral invasion and infection by binding to histo-blood group antigens (HBGAs), which is divided into two main domains: an internal shell domain (S region) and a protruding domain (P region), connected by a flexible hinge (Zhang et al., 2019).

Regarding the subtype of HuNoV, a dual typing system has been proposed for norovirus strains, due to frequent recombination at the ORF1/ORF2 sequences (Kapikian et al., 1972). According to the diversity of VP1, HuNoV can be classified into 10 genogroups (GI–GX) and at least 40 genotypes, with GI, GII, and GIV being most closely associated with human disease. Specifically, GII.4 strains have been responsible for the majority of outbreaks worldwide over the past 15 years (Vinjé, 2015). GII.4 variants emerge every 2 to 4 years, with new variants replacing previous GII.4 strains, leading to a rapid increase in cases over a short period until the next new genotype appears (Bull et al., 2010). Moreover, approximately 60 P-types have been described based on the nucleotide sequence of RdRp (Chhabra et al., 2019).

Shenzhen is a developed coastal city in China by high population mobility and a seafood diet. These factors likely contribute to the transregional migration and rapid transmission of various viruses, including noroviruses. Leveraging the sensitive surveillance system established in Shenzhen, we have conducted ongoing surveillance of various viruses, including noroviruses, over several years. This study focuses on the evolutionary characteristics and trends of noroviruses in Shenzhen from 2016 to 2022. First, we collected and sequenced the complete genomes of HuNoV from clinical samples between 2020 and 2022 and have contributed these sequences to online databases. In the context of global HuNoV sequences, and incorporating our previous sequencing results since 2016 (Hu et al., 2022), we systematically analyzed the genetic characteristics of HuNoV in Shenzhen. We conducted phylogenetic studies on HuNoV whole genome sequences and identified predominant variants. Meanwhile, Bayesian phylogenetic analysis of the Norovirus VP1 and RdRp regions revealed the common ancestor and temporal evolution characteristics of the dominant genotypes. Additionally, we evaluated the mutational effect on the structural stability of functional proteins in a dominant genotype. Moreover, we also investigated the recombination patterns of HuNoVs. Overall, this continuous surveillance and research provides valuable genomic data and offers new insights into the evolutionary study of noroviruses, which may promote the development of norovirus vaccines and enhance the prevention and control of gastroenteritis.

## 2 Materials and methods

### 2.1 Sample collection and preparation

From 2020 to 2022, hospitalized patients who met the case definition of diarrhea were enrolled in the sentinel monitoring hospitals of Shenzhen. Stool samples were collected and transported to the laboratory of the Shenzhen Center for Disease Control and Prevention (CDC), where they were stored at −80°C. According to the National Surveillance Protocol for Viral Diarrhea (2017 version), AGE was defined as three or more instances of loose stools within a 24-hour period, or fewer than three loose stools per day accompanied by symptoms such as vomiting, abdominal pain, fever, nausea, and dehydration, excluding the presence of pus or blood. This project was reviewed and approved by the Shenzhen Center for Disease Control and Prevention (Approval No.: SZCDC-IRB2024032). All samples were collected with the informed consent from the patients.

### 2.2 Nucleic acid extraction and reverse transcription

A 10% stool suspension of each sample was prepared using sterile phosphate-buffered saline and centrifuged at 8000 rpm for 5 minutes. Viral RNA was extracted from 200 µL of supernatant using a High Pure Viral RNA Kit (Roche) following the manufacturer’s instructions. The extracted RNA was stored at −20°C. All stool samples were tested for norovirus, rotavirus, sapovirus, enteric adenovirus, and astrovirus by real-time reverse transcription polymerase chain reaction (RT-PCR) using a rotavirus/norovirus (GI and GII) nucleic acid testing kit and a sapovirus/astrovirus/enteric adenovirus nucleic acid testing kit (Mabsky, Shenzhen, China).

### 2.3 Norovirus sequencing and genotyping

For norovirus-positive samples, extracted nucleotides were subjected to whole-genome sequencing. RNA libraries were constructed employing the U-mRNA seq Library Prep Kit for unbiased whole-genome sequencing, and sequencing was performed on the Illumina Novaseq 6000 platform with 150-bp paired-end reads. Raw FASTQ files were assessed with the FastQC tool v.0.11.9 and filtered using Trimmomatic v.0.39 (Bolger et al., 2014). The contigs were built with default parameters of the SPAdes program v.3.15.526 (Bankevich et al., 2012). Finally, the genomes of norovirus were assembled into complete genomic sequences using the Megahit software v1.2.9 (Li et al., 2016) and Geneious software v2023.2.1 (Kearse et al., 2012), respectively. Norovirus genotype was determined using an online genotyping tool (https://www.genomedetective.com/app/typingtool/nov/).

### 2.4 Spatial and temporal distribution of norovirus GII

Norovirus GII is the most common genotype causing disease in humans. To comprehensively understand the spatial and temporal distribution characteristics of norovirus GII strains in Shenzhen in recent years. We utilized ArcMap v10.8 (Redlands, CA: Environmental Systems Research Institute) to create a regional distribution map and analyzed the temporal distribution using Origin Lab v2022 (Northampton, MA, USA).

### 2.5 Phylogenetic analysis of norovirus

The whole genome sequences were used for phylogenetic analyses to determine the genetic characteristics of norovirus in the background of global strains. We utilized complete sequences from Shenzhen from 2016 to 2022, including our previous sequences (Hu et al., 2022) and sequences contributed in this study. Additional sequences were downloaded from the GenBank database (https://www.ncbi.nlm.nih.gov/genbank). Multiple sequences comparison were performed using MAFFT v7.505 (Katoh et al., 2002), and phylogenetic trees of the whole genome were constructed using the maximum likelihood method of IQ‐TREE v2.1.4,18 (Nguyen et al., 2015). Bootstrap values were calculated with 1000 pseudo-replicate data sets. The phylogenetic tree includes annotations for the location, date, and genotype of each strain.

To estimate the time to the most recent common ancestor (tMRCA) of human norovirus viruses, we first performed sequence deduplication on the MAFFT-aligned sequences. Then, using the reference sequence coding region as a standard, we separately extracted the VP1 and RdRp regions of Norovirus. Finally, we used Bayesian Markov chain Monte Carlo analyses for each gene in BEAST version 1.10.4 (Suchard et al., 2018) to generate two maximum clade credibility trees.

### 2.6 Evolutionary trends of dominant genotypes

For the dominant GII genotypes identified in the whole genome phylogenetic trees, we retrieved and downloaded the complete sequences of all such genotypes from GenBank to further investigate their evolutionary trends. Specifically, we used Origin software to analyze and map the annual distribution of these strains at three geographical levels: global, national (China, excluding Shenzhen), and regional (Shenzhen). The data for China does not include our contributed sequences from Shenzhen, as our sequences could be as extensive as those from other studies across China, thus potentially causing significant data skewness.

### 2.7 Mutational study in functional proteins for predominant strains

On the basis of previous studies, we inferred that a predominant and important genotype GII.4_Sydney[P31], is likely to exhibit periodic epidemic trends in Shenzhen. Accordingly, we explored its mutations on its functional protein VP1 to validate this conjecture. Using the GII.4_Sydney[P31] strain (Genbank Accession JX459908) as the reference sequence, we extracted the VP1 coding region from all GII.4_Sydney[P31] strains. These sequences were translated into amino acids using Bioedit software v7.2.5, allowing us to identify key residue sites for mutations.

Considering the variation among sequences, we analyzed the GII.4_Sydney[P31] sequences from Shenzhen over two periods: 2016–2018 and 2020–2022. We examined the amino acids at hotspot sites within these sequences and compared mutations between the reference sequence and the 2016–2018 sequences, as well as between the 2016–2018 and 2020–2022 sequences. We then evaluated the effect of these mutations on the structural stability of the GII.4 capsid protein (VP1). Specifically, we employed the FoldX v5.1 to evaluate quantitative changes in the gibbs energy of protein folding caused by these mutations (ΔΔG, unit: kcal/mol), on the P domain of VP1 protein in the GII.4_Sydney[P31] (PDB: 4WZT). In this method, a negative ΔΔG indicates stabilizing mutation while a positive ΔΔG indicates destabilizing mutation. Moreover, this protein has also been structurally visualized by Visual Molecular Dynamics (VMD) (Humphrey et al., 1996).

### 2.8 Recombination analysis

In this research, we conducted recombination analysis by the Recombination Detection Program version 4 (RDP4) v4.101 for identifying recombinant sequences and recombination breakpoints of norovirus strains. RDP4 was equipped with seven different computational models, including RDP, GeneConv, Bootscan, MaxChi, CHIMAERA, SisScan, and 3SEQ (Martin et al., 2015). Because these seven complementary models can mutually validate each other, recombination events were considered reliable when potential signals were detected by at least four of the models, with a p-value threshold of 0.05.

## 3 Results

### 3.1 Norovirus sequencing and genotyping

Among the norovirus-positive samples collected from 2020 to 2022, 172 complete genomes of norovirus were successfully sequenced using high-throughput sequencing. Genomic sequences were deposited into the NGDC database (https://ngdc.cncb.ac.cn/). Their corresponding clinical information, genotypes, and accession numbers are summarized (**Supplementary Table S1**).

**Figure 1:**
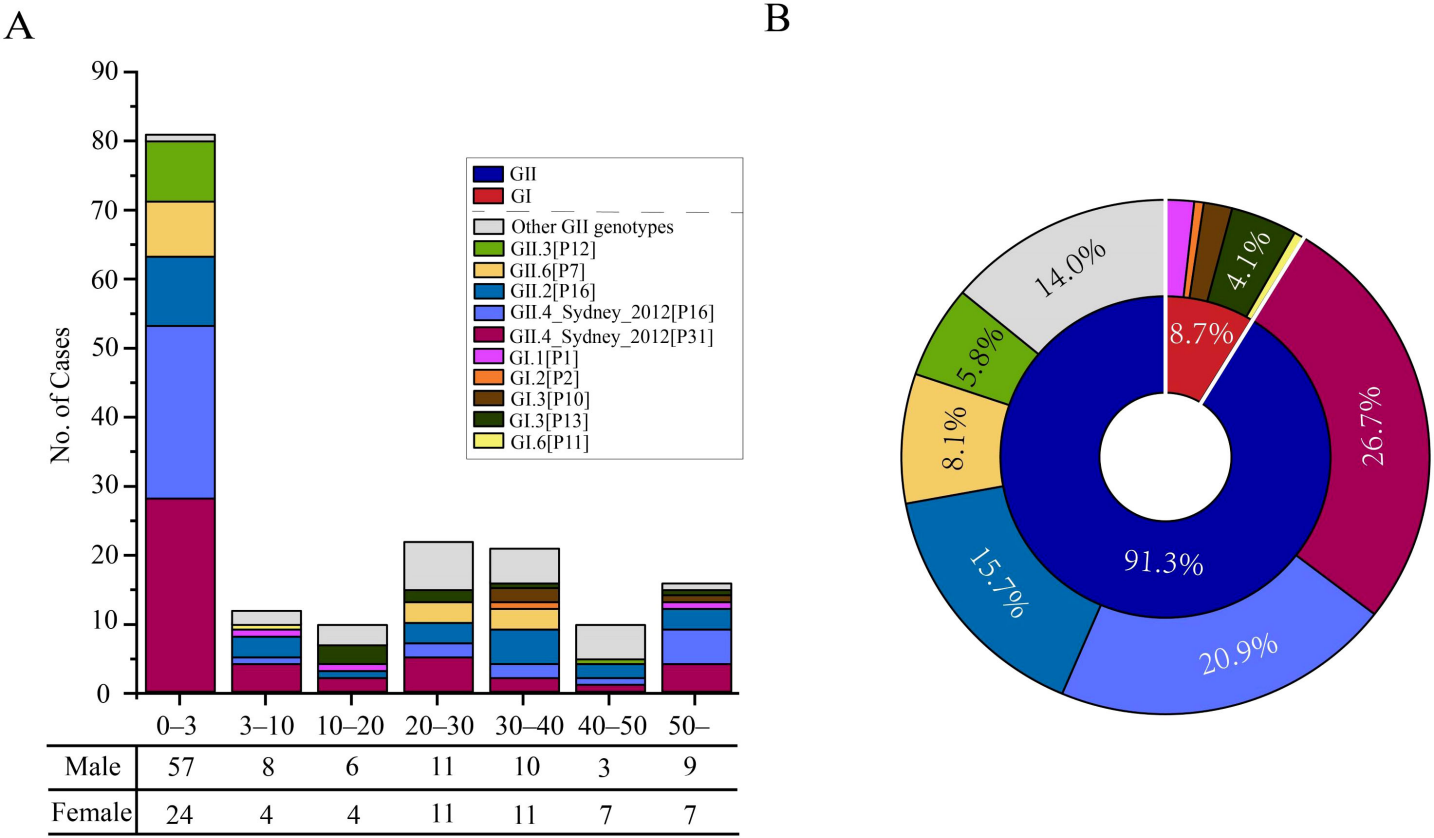
Demographic distribution of norovirus infection cases in Shenzhen from 2020 to 2022. **(A)** Distribution of genotypes in different age groups. The horizontal axes represent different age groups, where each age range includes the lower limit but excludes the upper limit. The table below the horizontal axis corresponds to the number of infection cases for males and females within different age groups. **(B)** Composition of genogroups and genotypes. The inner ring represents the genogroup, while the outer ring represents the gender.

We examined the age, gender, and genotypic distribution of infected individuals in Shenzhen to comprehend the demographic features of norovirus infections (**Figure 1**). Norovirus can infect people of all age groups, with respect to age distribution. However, compared to other age groups, the number of illnesses among children under 3 was noticeably higher (**Figure 1A**). This result was consistent with previous research showing that AGE in newborns and early children is largely caused by norovirus (Shah and Hall, 2018). Moreover, the incidence of infections was comparable in those between the ages of 20–30 and 30–40, second only to those under the age of three. This remains us that adult cases of norovirus also need to be a key focus in the subsequent epidemic prevention and control efforts in Shenzhen. Another susceptible group for norovirus is the elderly. In fact, the number of infected individuals over 50 years old was not significant in our study, with an infection rate higher than that of the 3–20 age group and the 40–50 age group, but lower than that of infants under 3 years old and adults aged 20–40 (**Figure 1A**). In addition to age distribution, the gender distribution has unique traits (**Figure 1A**). Generally, there were more male infections than female infections (60.5% versus 39.5%, respectively). Specific, male cases were about twice as high as female cases in the 0–10 age groups, while gender differences were minor in other age categories.

In terms of genotype, GII viruses were implicated in the majority of these cases, whereas GI viruses were responsible for a small number of cases (**Figure 1B**). Specifically, GII viruses accounted for 157 (91.3%) of the cases, while only 15 (8.7%) cases were infected by GI viruses. The prevalence of GII strains between 2020 and 2022 was in line with our earlier study, which was carried out between 2016 and 2018 (Hu et al., 2022). Within the GII strains, more than 10 distinct genotypes were identified, including 46 GII.4_Sydney[P31] (26.7%), 36 GII.4_Sydney[P16] (20.9%), 27 GII.2[P16] (15.7%), 14 GII.6[P7] (8.1%), 10 GII.3[P12] (5.8%) and other genotypes (**Figure 1B**). These genotypes were known to exist in the population of children under 3 years old. GII.3[P12] has also been detected in individuals aged 40–50, and GII.6[P7] has been found in the 20–40 age group as well (**Figure 1A**). For GI strains, only five genotypes were detected, mainly GI.3[P13] (4.1%), which were dispersed across different age groups.

### 3.2 Spatial and temporal distribution of norovirus GII

Sensitive surveillance systems have been implemented in Shenzhen to monitor the epidemiological distribution of norovirus. Based on this ongoing surveillance of norovirus for years, we investigated the dynamic distribution of norovirus cases from 2016 to 2022. Considering that GII strains comprised approximately 90% of cases, we focused our study on the specific distribution of GII strains.

**Figure 2:**
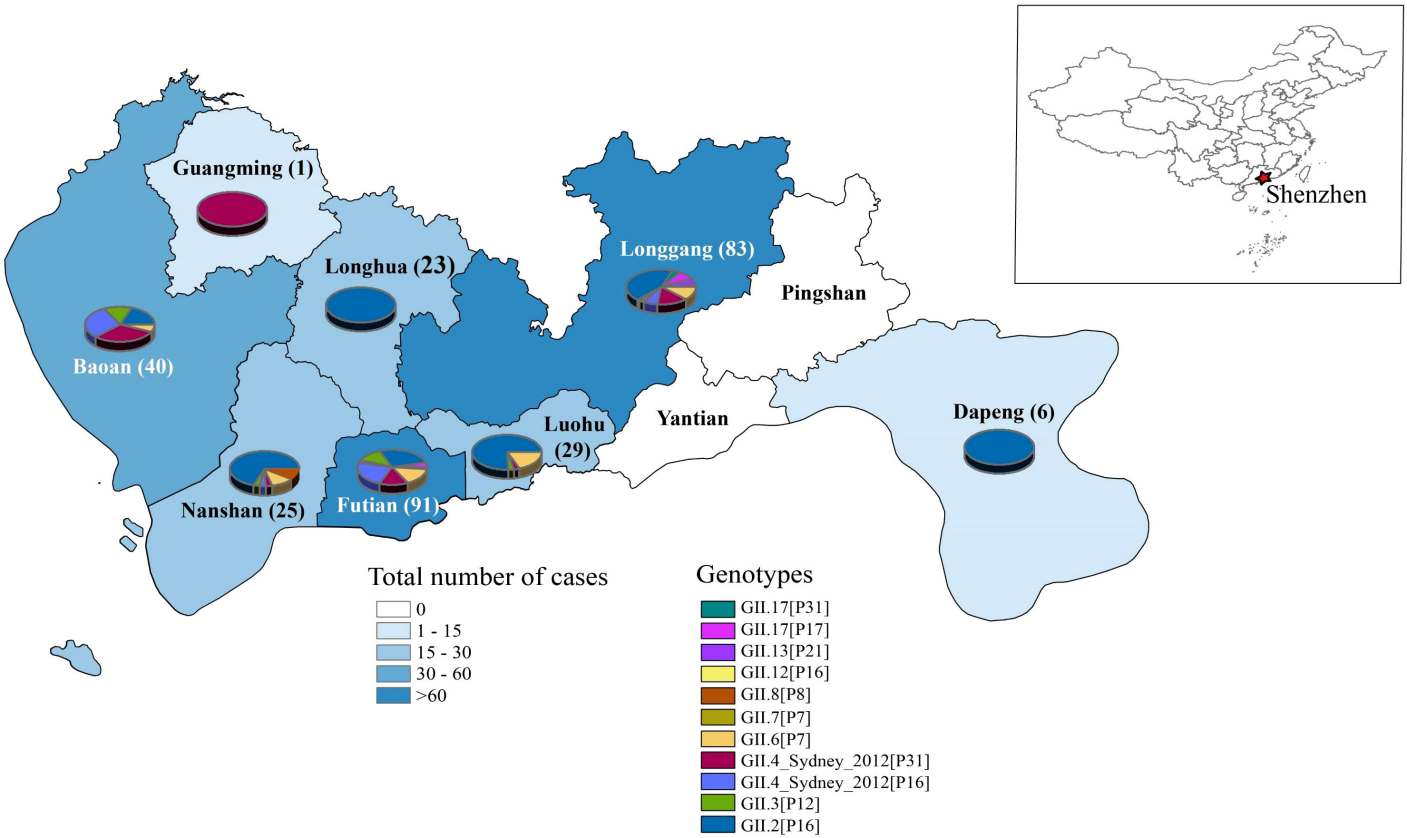
Geographical distribution of HuNOV GII in Shenzhen, 2016–2022. A map of China is shown to localize the studied city. The color of each region represents total number of cases and the numbers on the map denote the numbers of cases in the region. Different norovirus genotypes are indicated by color. The pie chart for each region indicates the composition of the genotype.

To investigate the regional distribution, we categorized norovirus strains based on their geographical information (**Figure 2**). Of ten administrative districts in Shenzhen, eight districts had reported for norovirus cases. The highest number of cases was concentrated in the Futian and Longgang districts, followed by Baoan and Luohu districts, while no cases were found in the Yiantian and Pingshan districts.

Besides the number of cases, it can be observed that Shenzhen has multiple genotypes co-circulating in 2016–2022 (**Figure 2**). For example, the GII.2[P16] genotype was one of the local predominant strains, which circulated in seven of ten regions in Shenzhen. Moreover, GII.4_Sydney[P31] and GII.4_Sydney[P16] were also relatively predominant genotypes, which were primarily distributed in the more populous and economically developed districts of Longgang, Futian, and Baoan. For other subtypes of norovirus, they were dispersed across multiple areas in Shenzhen and did not exhibit discernible characteristics.

In terms of the composition of the HuNoV genotype, different districts in Shenzhen demonstrated various patterns (**Figure 2**). Generally, Futian and Longgang, which reported the highest number of norovirus cases, also exhibited the most diverse and complex composition of norovirus genotypes. This may be attributed to their advanced economy and frequent population movements. In contrast, Baoan, Nanshan, and Luohu, although reporting fewer cases compared to Futian and Longgang, still experienced a relatively higher number of cases and displayed a comparatively complex genotype composition. For other districts, where reported cases were sporadic, the genotype composition tended to be simpler. An interesting exception is Longhua district, which reported over 20 norovirus cases all belonging to the same genotype (GII.2[P16]) and no other genotypes. This phenomenon may be attributed to several concentrated outbreaks of GII.2[P16], as evidenced by the close disease dates of these cases reported in our previous study (Hu et al., 2022).

**Figure 3:**
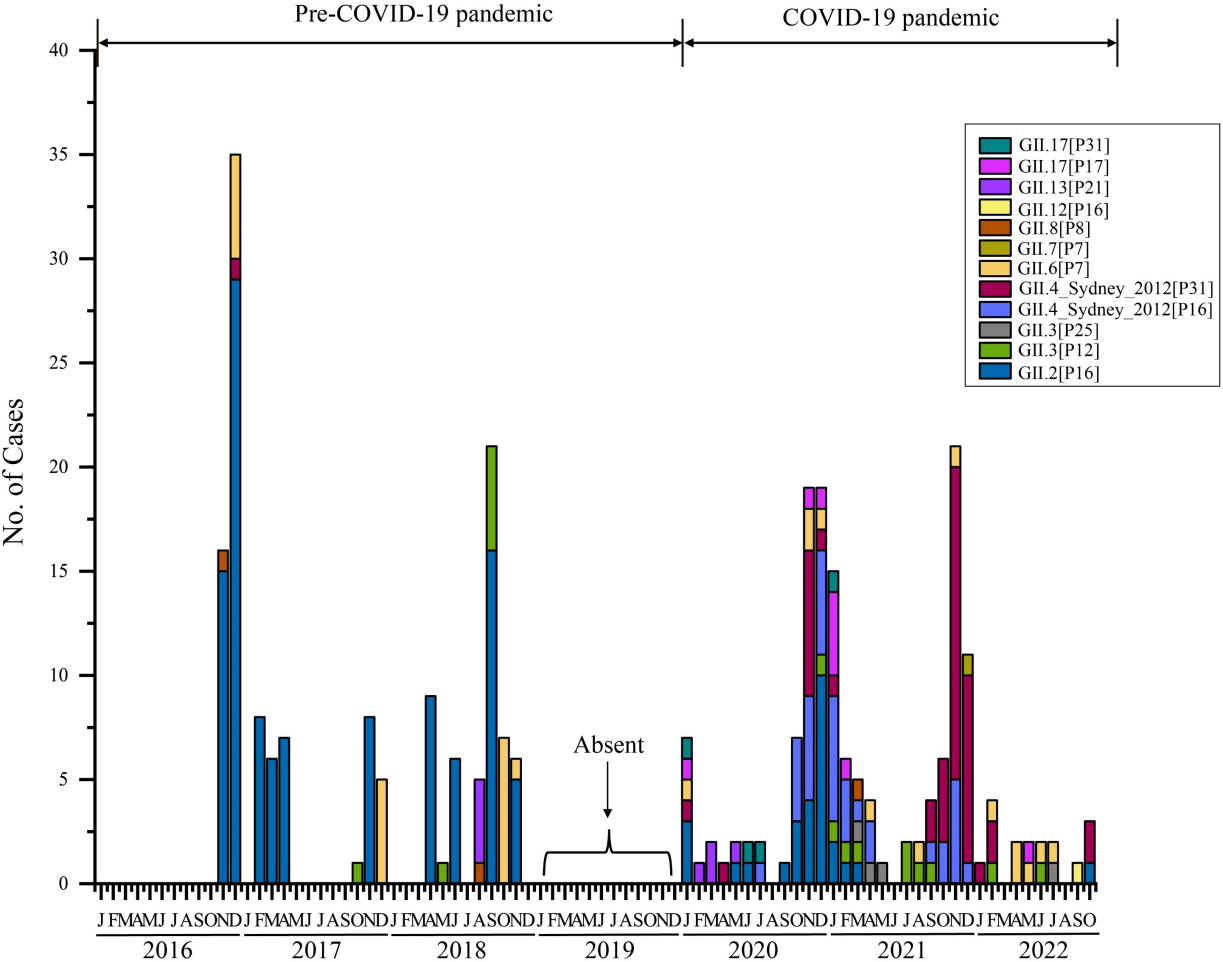
Temporal distribution of HuNOV GII in Shenzhen, 2016–2022. Different norovirus genotypes are indicated by color. Absent labels indicate period (2019) during which fecal sample collection was paused.

Furthermore, we analyzed the monthly distribution of norovirus GII in 2016–2022 (**Figure 3**). Overall, the prevalence of norovirus exhibits a distinct seasonal distribution. Typically, epidemics begin around August or September, peak towards the end of the year, and decline or disappear by March of the following year. Thus, norovirus outbreaks in Shenzhen primarily occur during the fall and winter seasons. In terms of genotypes, the proportions of each subtype varied by year (**Figure 3**). Historically, GII.4 genotypes were primarily responsible for the majority of norovirus epidemics worldwide before 2015 (Patel et al., 2009). However, our research revealed a new temporal distribution trend: GII.2[P16] became the most prevalent genotype in Shenzhen during 2016–2018, leading to persistent transmission of the AGE epidemic within Shenzhen. This pattern indicated that since 2016, norovirus strains in Shenzhen have differed from the globally predominant strains of previous years (GII.4). Another noteworthy observation is the significant change in norovirus genotype composition in Shenzhen before and after the COVID-19 pandemic. Specifically, since the onset of the pandemic at the end of 2019, circulating norovirus strains have shifted from predominately GII.2 strain to various genotypes, with a majority being GII.4_Sydney[P16] and GII.4_Sydney[P31] during 2020–2022. In 2020, the circulation of GII.2 had already decreased, although it still accounted for a notable proportion of cases. By 2022, the GII.2 strain had virtually disappeared and was replaced by GII.4 stains, particularly GII.4_Sydney[P31], which became predominant then (**Figure 3**).

### 3.3 Phylogenetic analysis of norovirus

**Figure 4:**
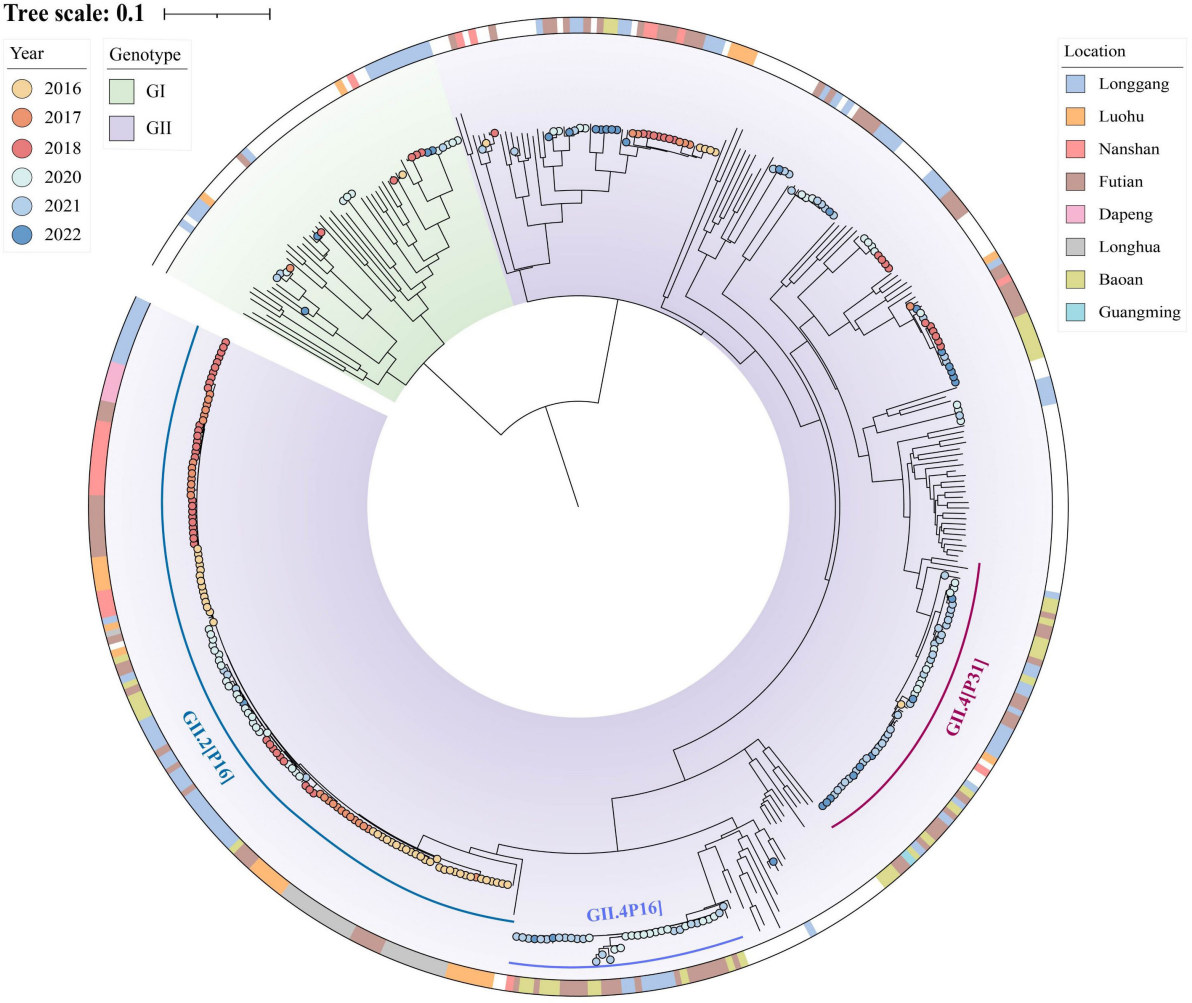
Phylogenetic tree of human norovirus (HuNoV). The background color represents the viral genotype. The color strip depicts the location and the color of solid circle represents the time. The sequences annotated with time and location information are the strains contributed in current or previous study. Other sequences can be downloaded from the GenBank database, with accession numbers shown in the figure. The three dominant genotypes are marked by three arcs of different colors.

In the context of global sequences, we conducted whole genome phylogenetic analysis of HuNoV sequences from Shenzhen covering the period from 2016 to 2022 (**Figure 4**). This analysis included both newly sequenced strains from 2020 to 2022 and previously sequenced strains (Hu et al., 2022). Meanwhile, a Bayesian phylogenetic analysis of the norovirus VP1 region and RdRp region was conducted to reveal the origin of norovirus and the temporal changes of the dominant genotypes.

The maximum-likelihood phylogenetic tree of full-length norovirus sequences consists of two genetic groups: GI and GII (**Figure 4**). Generally, GII comprised more than half of the tree, consistent with a previous report that GII was the predominant circulating norovirus globally (Lun et al., 2018). The GII genotype can be further divided into multiple subgroups, in which we identified over ten distinct subtypes. On the contrary, GI has persisted in a sporadic form for years and appears to have fewer subtypes compared to GII (**Figure 4**).

Furthermore, we concentrated on the evolutionary characteristics of GII in Shenzhen and observed several dominant subtypes with different evolutionary features (**Figure 4**). First, the GII.2[P16] genotype had the largest number of sequences in Shenzhen and clustered in a single branch. Specifically, GII.2[P16] has increased rapidly in Shenzhen since 2016, but gradually declined after 2020. In addition to GII.2[P16], the new variant GII.4_Sydney[P16] exhibited an independent evolutionary trajectory, which was phylogenetically close to a strain (MT029316) reported in the United States in 2018. This variant started its prevalence in Shenzhen in 2020 and circulated widely over the following two years. Another notable genotype is GII.4_Sydney[P31]. Only one GII.4_Sydney[P31] strain was collected in 2016, but it re-emerged in 2020 and caused a significant epidemic in several districts of Shenzhen (**Figure 4**).

We further traced the origin of the norovirus. In the analysis of the Bayesian phylogenetic tree of the norovirus VP1 region (**Supplementary Figure S1**), we found that the origin of the norovirus VP1 protein can be traced back to Japan between 500 and 550 AD. At the same time, the common ancestor of the GI and GII genomes also originated from Japan. The study indicates that the norovirus strains in Shenzhen primarily formed through three modes of introduction. The first mode is through introduction from Japan to China, which is the main source of norovirus strains in Shenzhen, such as the dominant genotype GII.2, which was likely introduced from Japan to Shenzhen after 1935. Another mode of introduction is from the United States, with the GII.4 strains in Shenzhen most likely introduced from the United States. after 1940. Additionally, some strains exhibit a pattern of internal circulation within China. Regarding the evolutionary history of the norovirus RdRp region (**Supplementary Figure S2**), the norovirus RdRp region originated in Japan between 900 and 950 AD. Similarly, the three modes of transmission mentioned above are also observed. The dominant genotype GII.P31 in Shenzhen was likely introduced from the United States after 1960, while the common ancestor of GII.P16 in Shenzhen may have originated in Japan around 1950.

### 3.4 Evolutionary trends of important genotypes

**Figure 5:**
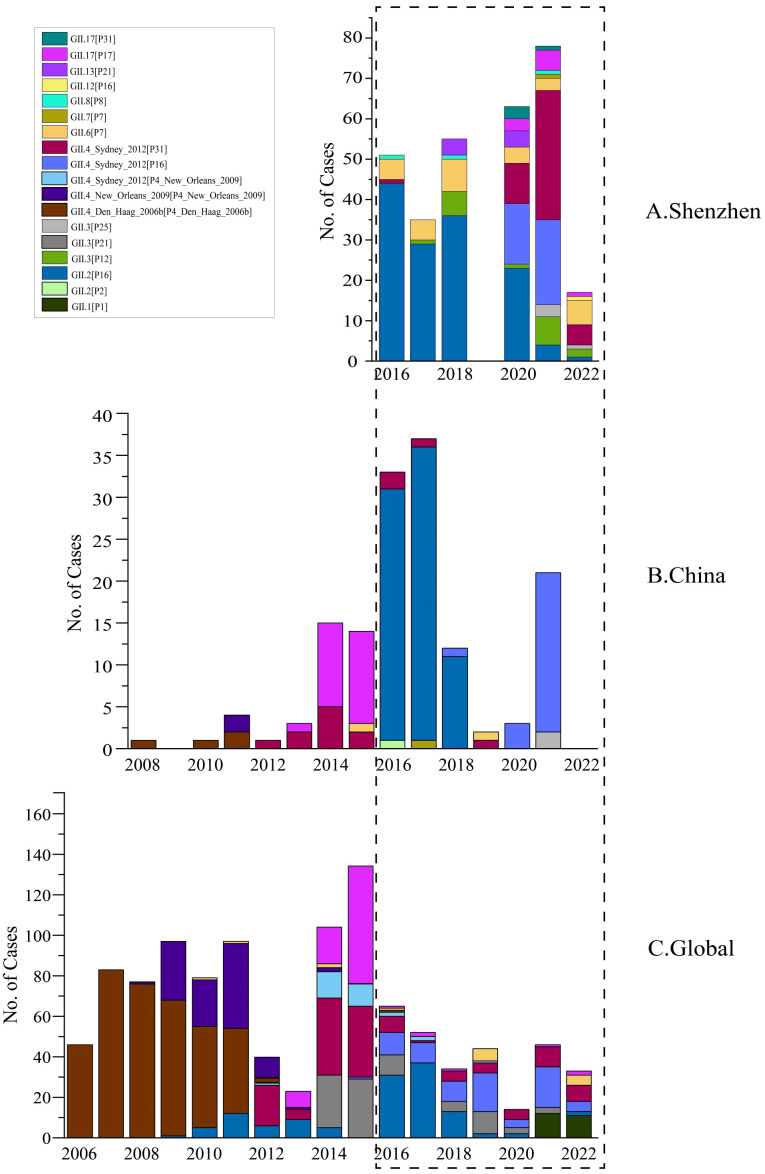
Temporal distribution of HuNOV GII genotypes in Shenzhen **(A)**, in China **(B)**, in Global **(C)**. Different norovirus genotypes are indicated by color. Blank areas indicate that sequences related to that year were not collected or downloaded.

Based on those observed dominant strains, we further compared their dynamics of genotype percentages across different geographic levels (global, national, and regional) to investigate their evolutionary trends (**Figure 5**). The GII.2[P16] strain, first emerged in 2009 and was then introduced into China in 2016. Since then, this genotype has dominated in Shenzhen and other cities in China, before its decline in 2020. Another strain, GII.4_Sydney[P16], which emerged in 2016, was first reported in China in 2018 and subsequently caused a pandemic in 2020. In terms of emergence and diffusion, the transmission pattern of both GII.2[P16] and GII.4_Sydney[P16], can be similar, and their transmission trends are mostly consistent in different geographical levels. Both genotypes emerged outside China, were subsequently introduced into Shenzhen, and circulated there for several years before declining. The only difference is that GII.4_Sydney[P16] has not yet displayed clear delineation. On the contrary, the GII.4_Sydney[P31] strain exhibited different evolutionary patterns. In the global level, GII.4_Sydney[P31] demonstrated a periodic pattern. Initially, it had been circulating globally from 2012 to 2015. Its prevalence was then supplanted by other genotypes from 2016 to 2018, with only a few cases reported, but it re-emerged and became one of the circulating genotypes globally. In China, excluding Shenzhen, this periodic pattern was not apparent. The GII.4_Sydney[P31] genotype declined after 2016 and remained a minority without significant resurgence. Regarding Shenzhen, a vast increase in GII.4_Sydney[P31] was observed after 2020. Due to the absence of data before 2016, the post-2016 decline of GII.4 _Sydney[P31] in Shenzhen could not be directly observed. Nevertheless, based on data from China and global sources, it is reasonable to assume the existence of such a decline in 2016 and to infer that a periodic pattern likely exists.

### 3.5 Analysis of amino acid variation within GII.4 capsid sequences

Antigenic drift is caused by amino acid changes within the capsid protein (VP1), particularly within the protruding P2 domain, which enables the virus to escape from population host immunity (Shanker et al., 2011). We assumed the existence of a decline of GII.4_Sydney[P31] in Shenzhen and inferred that a periodic pattern of GII.4_Sydney[P31] likely existed in Shenzhen. To validate the potential decline in 2016–2018 and explain the observed increase or resurgence during 2020–2022, we examine the mutations in the functional protein VP1 for supporting evidence. We extracted amino acid sequences of GII.4_Sydney[P31] VP1 and identified important mutational sites. We subsequently investigated mutations associated with the potential decline and resurgence of this genotype by evaluating their effects on the protein stability of VP1.

**Figure 6:**
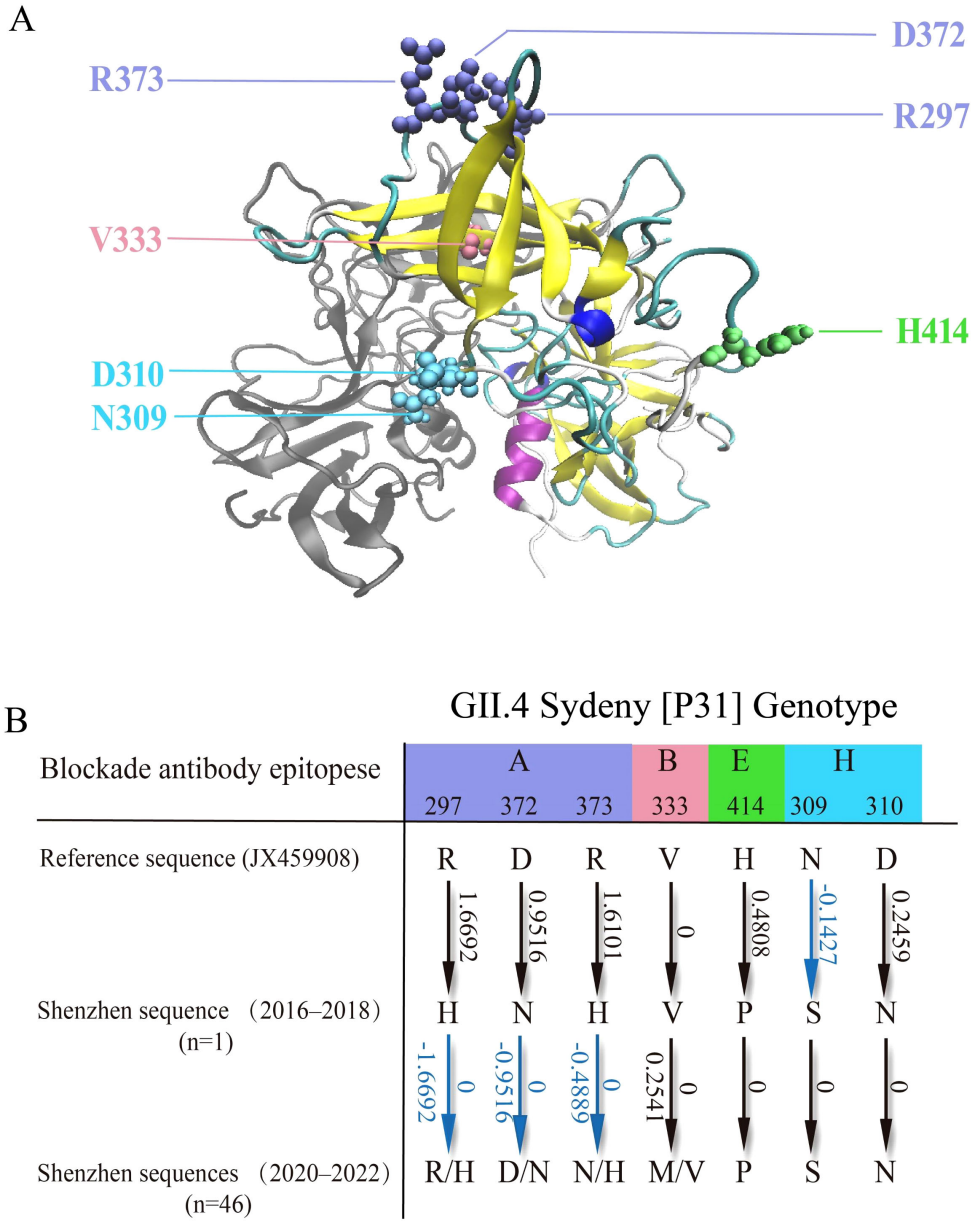
Structural study of key mutations in the GII.4_Sydney[P31] P Domain of VP1. **(A)**. The visualized structure of the P domain of VP1 (PDB: 4WZT). Key mutational sites are denoted by colored spheres, with each color corresponding to a specific blockade antibody epitope. **(B)**. Mutational analysis of GII.4_Sydney[P31] sequences in Shenzhen, focusing on blockade antibody epitopes and their effects on VP1 stability. Amino acid mutations are depicted by arrows, with corresponding changes in gibbs free energy (ΔΔG, kcal/mol). Stabilizing effects (ΔΔG < 0) are shown in blue. Multiple mutation directions are separated by slashes.

We identified seven key mutation sites in GII.4_Sydney[P31] VP1 sequences from Shenzhen, most of which are associated with viral escape and evolution (Debbink et al., 2012). These mutation sites were mapped and visually represented within the three-dimensional structure (**Figure 6A**). Based on relevant literature (Zheng et al., 2022), we identified the blockade antibody epitopes associated with each mutational site (**Figures 6A, B**). Specifically, mutations were found at residue sites 297, 372, and 373 in epitope A; residue site 333 in epitope B; residue site 414 in epitope E; and residue sites 309 and 310 in epitope H. These residue sites and their associated mutations may be related to the viral immune escape of GII.4_Sydney[P31].

We further identified mutations at these hotspot sites by comparing the reference sequence of GII.4_Sydney[P31] (JX459908) and the sequences from 2016 to 2018 sequences, as well as between comparing the sequences from 2016 to 2018 with those from 2020 to 2022. Subsequently, we utilized FoldX to evaluate their mutational effects on the stability of the VP1 protein (**Figure 6B**). For the 2016–2018 sequence, most mutations (except N309S mutation) led to positive changes in gibbs free energy (ΔΔG). Consequently, these mutations resulted in an overall increase in ΔΔG, potentially destabilizing the VP1 protein. This destabilization may contribute to the limited number of cases of this genotype in Shenzhen during 2016–2018 (**Figure 4**), as well as the potential decline of this strain. However, the situation differed with sequences from Shenzhen between 2020 and 2022. Specifically, for these hotspot sites, some sequences evolved through mutations, either reverting to the previous reference (JX459908) or developing entirely new mutations (**Figure 6B**), which may stabilize the VP1 protein. This phenomenon aligns with our previous observation of an increase in GII.4_Sydney[P31] during 2020–2022, particularly in Shenzhen.

### 3.6 Recombination analysis

In addition to mutations, recombination events also play a significant role in the evolution of norovirus. This section investigated recombination events among norovirus strains and explored the patterns and mechanisms of recombination.

We summarized the recombination events detected by multiple method of RDP4 (**Supplementary Table S2**). The complete sequence of all norovirus has undergone 21 recombination events (**Supplementary Table S2**). All recombination events occurred within the same genogroup, either GI or GII, with no recombination observed between GI and GII. Among these events, the majority were within GII (16 events), which is significantly higher than the number of events with GI (5 events). Specifically, three recombination events (#14, #15, #16) occurred within the same genotype, while the other events took place between different genotypes. In certain specific events (#6 and #13), the recombinant strains and corresponding two parental strains can be categorized into three distinct genotypes, making the recombination process complex with regard to the genotypes. Furthermore, an interesting observation was that a strain from Shenzhen (C_AA085029.1) experienced three recombination events at distinct locations, namely #13, #17, and #18. Of these, two recombination events (#13 and #17) involved identical parental sequences, whereas the third recombination event (#18) was associated with a different minor parent.

In terms of recombination fragments and breakpoints, the positions of these fragments and breakpoints exhibit certain patterns (**Supplementary Table S2**). The lengths of recombinant fragments can be various, ranging from approximately 200 bp to 2600 bp. The majority of recombinant fragments (11 out of 21) are around 2400 bp long, nearly covering the entire ORF2 and ORF3. Other recombination segments mainly occurred within ORF1 or ORF2, as well as across the ORF boundary. Regarding recombinational breakpoints, more than half (13 out of 21) recombination events originated from positions between approximately 4900 bp and 5200 bp, corresponding to the junction of ORF1 and ORF2. Most of these, recombination breakpoints end at the sequence termini, indicating that nearly the structural genomic region (ORF2 and ORF3) underwent entire segmental exchange with the parental sequence, resulting in new genetic characteristics. Other recombination breakpoints were distributed within ORF1 (5 out of 21) and ORF2 (3 out of 21). Therefore, the 300 bp region (between 4900 bp and 5200 bp) can be a recombination-prone region, accounting for over half of the observed events. Within this region, the breakpoints are closely clustered, suggesting that a common feature in this area shared across various genotypes. This phenomenon may be associated with the evolutionary dynamics of noroviruses.

**Figure 7:**
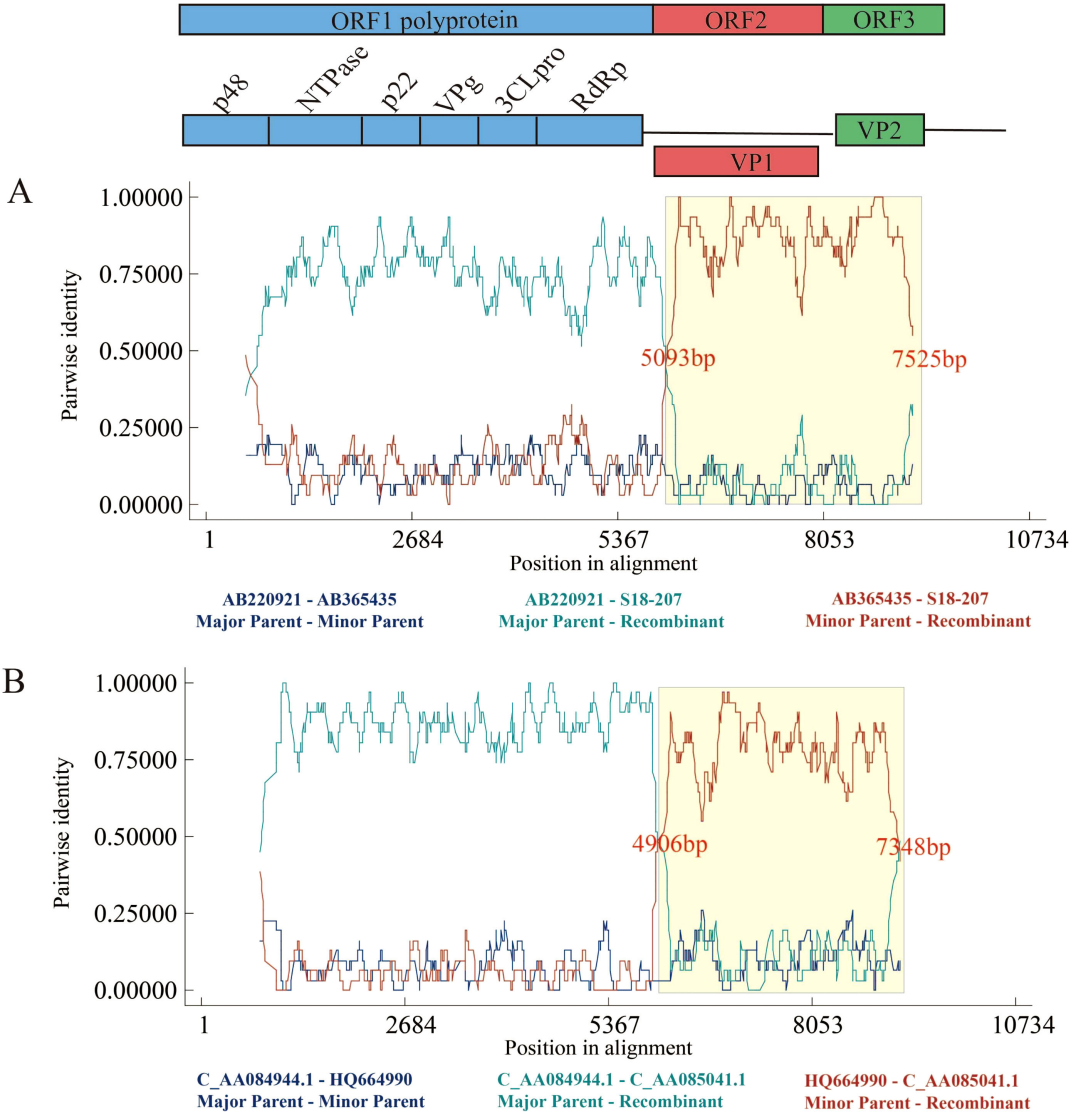
RDP results of recombination events including the sequences identified in the present study. **(A)** Event #8. **(B)** Event #9. Recombination events are numbered as in **Supplementary Table S2**. The colored lines represent the pairwise similarity between sequences.

Regarding the recombination in our contributed strains from Shenzhen, there are 17 recombination events associated with these sequences, namely #1 and #6–21 (**Supplementary Table S2**). Of these, we selected and presented the RDP results of events #8 and #9 (**Figure 7**), in which our Shenzhen strains were identified as recombinant strains, with recombination occurring at approximately 5000–7000bp. One recombinant strain (event #8) was the Shenzhen strain GII.3[P12] from 2018, with a Japanese strain serving as its primary parent and carrying the GII.4[P12] genotype. Additionally, the US norovirus strain GII.3[P21] was the secondary parent of it. The other recombinant strain (event #9) from Shenzhen in 2022 was produced by recombination exchange between the US strain GII.12[P33] from 2010 and the Shenzhen strain GII.2[P16] from 2020. Another interesting phenomenon is that, the recombination breakpoints of event #8 started at 5093 bp and ended at 7525 bp, while that of event #9 began at 4906 bp and ended at 7348 bp. In other words, their recombination fragments are similar, with breakpoint starting near the junction of ORF1 and ORF2 and extending to the terminus of ORF3. Consequently, these two recombinant strains were also consistent with the recombination-prone region observed in this study.

## 4 Discussion

Rotavirus, norovirus, astrovirus, sapovirus, and enteric adenovirus are the main viral pathogens accounting for more than 80% of viral acute gastroenteritis cases. With the development of global rotavirus vaccines, disease resulting from rotavirus infection has decreased, but the infection of norovirus still persists. At present, norovirus has become one of the most common causes of pediatric gastroenteritis cases (Lopman et al., 2016). In China, high population density and frequent communications from different regions and countries may also contribute to the rapid transmission of HuNoV. Thus, it is vital to enhance surveillance efforts and comprehend the evolutionary pattern of HuNoV, for the development of drugs and vaccines to prevent and control potential epidemics. Leveraging the sensitive surveillance system established in Shenzhen in Southern China, we carried out continuous surveillance of noroviruses over the years. We collected and sequenced the complete genomes of HuNoV from clinical samples and investigated the dynamic of infection norovirus and explored their evolutionary characteristics and mechanisms.

Identification of populations at risk of infection and transmission are always crucial for disease control. From a population-based HuNoV AGE monitoring, age and gender distribution of norovirus from Shenzhen between 2020 and 2022 was determined. Our research indicated that all age groups are susceptible to norovirus. Specifically, children aged three years and younger are the primary infected group, accounting for approximately half of all cases (**Figure 1A**). Children under the ages of three have the highest risk of contracting norovirus gastroenteritis, according to a consistent body of literature (Zhou et al., 2020; Pazdiora et al., 2022). This observation could be explained by the limited development of gastrointestinal function in infants, exposure to sensitive settings, and immature immune systems. Additionally, we have also focused on a different demographic segment: individuals aged 20 to 40. The incidence of infection in this group in Shenzhen is higher than that in individuals over 50, although it remains lower than that in children under three (**Figure 1A**). Meanwhile, the infection rate among the elderly population in Shenzhen was relatively low. These phenomena may be related to the dietary culture and demographic structure in Shenzhen, where young adults constitute a larger proportion of population compared to other age groups.

Regarding the viral types of noroviruses in Shenzhen, over 90% of norovirus infections were caused by GII, which accounts for a considerably greater number of cases than GI.

There are significant differences in the number of infected individuals between the two genetic groups, which makes GII the focus of research and the main target for prevention efforts in recent years. In terms of GII genotype composition, there were several genotypes circulating in Shenzhen. The most frequently detected genotypes were GII.4_Sydney[P31] and GII.4_Sydney[P16], accounting for 26.7% and 20.9%, respectively, followed by GII.2[P16], GII.6[P7], and GII.3[P12]. Notably, GII.4 infections represent nearly half of all norovirus cases in Shenzhen and has also been responsible for the majority of gastroenteritis infections worldwide, which reminded us the need for additional attention and prevention for GII.4. In contrast, the limited number of GI subtypes resulted in their scattered distribution in Shenzhen (**Figure 1B**). These genotypes also showed varying proportions of infections across different age groups, with multiple GII genotypes identified in children under three years old, while the genotype composition was relatively simple in other age categories (**Figure 1A**). This variation in susceptibility among age groups to different norovirus genotypes warrants further investigation. Accordingly, the investigation of demographic distribution can provide a reference for subsequent monitoring efforts and targeted prevention and control measures.

Our investigation of norovirus infections in Shenzhen from 2016 to 2022 revealed significant regional distribution patterns (**Figure 2**). We found a clear correlation between the number of infections in different regions and the abundance of viral genotypes: regions with a higher number of reported cases exhibited greater genotype diversity. The majority of cases were clustered in Longgang, Futian, and Baoan districts, accounting for over 75% of GII cases reported from 2016–2022 (**Figure 2**). Among these, the region with the highest abundance of viral genotypes was Longgang district, followed by Futian district, and finally Baoan district. This phenomenon may be attributed to several factors, including the developed economy, frequent population mobility, and advanced healthcare infrastructure in these districts. In regions with more developed economies and significant population mobility, conditions are more favorable for the transmission and spread of viruses, resulting in more frequent outbreaks and a wider variety of genotypes. On the contrary, Luohu and Nanshan had fewer recorded cases compared to Longgang, Futian, and Baoan, with only over 20 instances of norovirus AGE identified. Despite this, the genotypes of the norovirus also showed some abundance from Luohu and Nanshan districts. Additionally, Dapeng and Guangming districts have only recorded occurrences, while Pingshan and Yantian districts have not reported any cases. An exception was Longhua district, which had more than 20 cases identified, but only a single genotype, GII.2[P16], was found. This suggested a possible outbreak event linked to GII.2[P16], as evidenced by the temporal data gathered from samples collected in the area (**Supplementary Table S1**).

Norovirus gastroenteritis was typically described as *the winter vomiting disease* in history (Rydell et al., 2011). Regarding the temporal distributions, norovirus incidents typically begin around August or September in Shenzhen, peak in December or January, and decline or disappear by March of the following year (**Figure 3**). Thus, norovirus outbreaks in Shenzhen primarily occur during the fall and winter seasons, which aligns with other reports (Kitajima et al., 2010; Giammanco et al., 2017). Moreover, our observation also demonstrate that norovirus cases can occur in late summer (**Figure 3**), which has been noted in other regions of China (Zhu et al., 2021). Therefore, the activity of norovirus may be correlated seasonal changes and temperature, which has also been supported by the Pearson correlation analysis (Ouedraogo et al., 2017).

Phylogenetic analysis revealed the genetic diversity among noroviruses in Shenzhen (**Figure 4**). Among various genotypes of GII, one important subtype is GII.2[P16], which has been the most frequently detected subtype in our ongoing surveillance work. As one of dominant genotypes, it led to the continuous epidemic transmission in Shenzhen during 2016 to 2020, and its prevalence began to decline in 2020. Another significant genotype is GII.4, which includes the subtypes GII.4_Sydney[P16] and GII.4_Sydney[P31], these two genotypes exhibited diverse transmission trends at different geographical levels (**Figure 5**). The literature has shown that GII.4 generates new variants approximately 2 to 4 years by antigenic drift or shift, supplanting previous predominant strains (Bull et al., 2010). This phenomenon can be also observed in our ongoing study (**Figure 5**). In global and China (excluding Shenzhen), GII.4_Sydney[P31] displayed a clear trend of initial increase followed by a decrease. In the following years, another genotype, GII.4_Sydney[P16], exhibited a similar trend in global. In China (excluding Shenzhen), GII.4_Sydney[P16] had disappeared by 2022, but the specific trends remain to be investigated after 2022. However, when focusing on Shenzhen, these two genotypes appeared to circulate in 2020 simultaneously without a discernible sequential relationship. Therefore, while new variants of GII.4 caused viral epidemics every few years, this perspective was characterized by regional specificity in Shenzhen. This highlighted the importance and significance of ongoing pathogen surveillance in Shenzhen.

Building on the findings of the Bayesian phylogenetic analysis, we further traced the origin of norovirus to gain deeper insights into its evolutionary history. Both the norovirus VP1 region and the RdRp region have a common origin from Japan, involving three modes of transmission: introduction from Japan to China, introduction from the United States to China, and internal transmission within China. The dominant genotypes in this study mainly originated from the United States and Japan. This process reveals the complex evolutionary history and transmission pathways of norovirus in Shenzhen, indicating that virus transmission is complex and unpredictable in the context of globalization.

In this research, we compared the evolutionary trends of different genotypes across geographical levels, focusing on their prevalence and transmission patterns (**Figure 5**). For instance, GII.2[P16] generally exhibited a consistent pattern of initial growth followed by a subsequent decline across three geographical levels. This trend indicated a potential cycle of outbreaks that may require targeted public health responses. Another important genotype, GII.4_Sydney[P16], demonstrated an increase starting in 2016, with a decline observed from 2019 to 2020 across the globe. In China, this genotype was first reported in 2018, experiencing a peak in prevalence in 2021. This genotype also emerged as one of the dominant genotypes during 2020–2021 in Shenzhen, but it significantly decreased in 2022. However, the trajectory of GII.4_Sydney[P16] after 2021 in China (except for Shenzhen) remained uncertain, necessitating ongoing monitoring to determine whether it will follow the decline observed globally and within Shenzhen. Interestingly, we also identified other genotypes, such as GII.6[P7] and GII.3[P12], which exhibited distinct evolutionary patterns (**Figure 5**). For GII.6[P7], it followed a similar pattern of initial increase and subsequent decrease in Shenzhen from 2016 to 2022, while other countries and cities only reported sporadic cases of this genotype. Furthermore, there have been no documented cases of the genotype GII.3[P12] worldwide or in China (except for Shenzhen), but it exhibited an evolutionary trend of initial increase followed by decrease in Shenzhen, causing localized transmission between 2017 and 2022. Compared to GII.2[P16] and GII.4_Sydney[P16], the evolutionary patterns of GII.6[P7] and GII.3[P12] appear to be distinct in Shenzhen, reflecting trends specific to this region rather than national or global levels. Therefore, different genotypes may have diverse evolutionary trends in geographical levels, underscoring the necessity for ongoing surveillance in the future.

Specifically, we observed an interesting genotype the GII.4_Sydney[P31]. Globally, GII.4_Sydney[P31] expanded rapidly after 2013, began to decline in 2015, but showed signs of resurgence from 2018. Similarly in China (excluding Shenzhen), the GII.4_Sydney[P31] strain rose from 2012 and then decreased from 2014. In Shenzhen, however, due to the shortage of sample data, we cannot observe its cases prior to 2015 in Shenzhen. We only observed a significant rise in GII.4_Sydney[P31] cases since 2020. Consequently, we have not yet identified a cyclical pattern of emergence, decline, and resurgence for GII.4_Sydney[P31] in Shenzhen. According to related research, however, Shenzhen experienced an outbreak of GII.4_Sydney[P31] in 2012 (He et al., 2016). Meanwhile, our observation that only one case of GII.4_Sydney[P31] was found from 2016 to 2018, which indicates a potential decrease. Subsequently, GII.4_Sydney[P31] was most prominent from 2020 to 2022, demonstrating an increase or resurgence after decrease. As a result, based on relevant literature and considering the phenomenon of a decline in GII.4_Sydney[P31] at the other geographic levels in 2016, it is reasonable to hypothesize that GII.4_Sydney[P31] in Shenzhen also underwent similar cyclical patterns of epidemic rise, decline, and resurgence.

Taking into account the possibility of such cyclical patterns, we analyzed and verified this trend by investigating amino acid mutations in the VP1 protein. Mutations may cause stability alterations in VP1, a critical structural protein involved in the formation of virus capsids, which could impact functions and viral adaptability of VP1. We identified seven mutation sites within the P2 sub-domain epitope of GII.4_Sydney[P31] VP1 in Shenzhen (**Figure 6A**), and documented specific mutations occurring at these important sites between 2016–2018 and 2020–2022. On one hand, from the perspective of antigenic epitopes, norovirus can lead to epidemics by escaping the pressure of population immunity due to alterations in these epitope area (Donaldson et al., 2010; Lindesmith et al., 2013). These mutations may affect viral fitness and epidemic potential, thereby supporting our hypothesis regarding the fluctuating prevalence of norovirus in Shenzhen. On the other hand, the evaluation of the VP1 protein stability through FoldX showed that, the mutations from 2016–2018 sequences can destabilize the VP1 protein, whereas the mutations from 2020–2022 sequences can stabilize the protein (**Figure 6B**). In summary, the analysis of antigenic sites and the stability of VP1 can provide an explanation for the hypothesis that GII.4_Sydney[P31] in Shenzhen may have a trend of rise, decline, and resurgence.

Another crucial factor in the evolution of norovirus is recombination (**Figure 7**). Recombination enhances genetic fitness of the virus, facilitates its evolution, and enables it to spread within host populations by evading the host immune response (Pérez-Losada et al., 2015). In our study, we examined the recombination characteristics of noroviruses. Our research found that recombination occurred exclusively within the GI and GII genogroup, but it was able to take place between different subtypes. This allows for the exchange of genetic material among different subtypes, leading to the formation of new genotypes. These newly formed genotypes could affect the virulence, transmission efficiency, and ability to evade the host immune response. Additionally, some recombinant strains underwent multiple recombination events at different locations, indicating that the evolutionary process of norovirus can be complex and diverse. This complexity may enable the virus to rapidly adapt to changing environments and host immune responses. Therefore, it is crucial to conduct in-depth research on the molecular mechanisms of recombination to understand the genetic dynamics of norovirus.

In terms of recombination breakpoints, most starting breakpoints are concentrated in a specific region (from 4900 to 5200bp), partially overlapped with the RdRp (3568–5097 bp) and ORF2 (5081–6703 bp). This recombination-prone region is consistent with the previously reported locations of recombination breakpoints (Bull et al., 2005; Tohma et al., 2021; Chen et al., 2023). When it comes to the terminating breakpoints, the majority of them are found at the 3’ end of ORF3, indicating that almost the entire ORF2 and ORF3 have been exchanged between parental strains. In this instance, the recombinant section makes up almost 30% of the entire genome length and is fairly long (approximately 2400 bp). Extensive genetic recombination has the ability to confer novel traits or adaptations on the recombinant strain, which could potentially result in significant modifications to its pathogenicity and transmission. Moreover, previous studies have revealed that recombination at this region allows the virus to exchange its viral capsid while retaining the region involved in viral genome replication (White, 2014). Consequently, this mechanism enables the norovirus to adapt more effectively to immune responses while maintaining essential functions for replication.

As a single-stranded RNA virus, norovirus is highly unstable. This instability makes it prone to mutations and recombination, which in turn can affect the rate and severity of infections in the population. The limitation of this study is the missing or incomplete clinical information from some sentinel hospitals, which prevents us from conducting an in-depth analysis of the correlation between genotype changes and clinical manifestations, and from fully assessing the potential impact of new genotypes on disease severity and transmissibility. Consequently, there remains much to be discovered in terms of explore the evolutionary mechanisms of norovirus and its potential impact on public health. Even so, our study still provided an in-depth and targeted investigation and analysis of the evolutionary characteristics of the norovirus. On this basis, a transmission trend of the dominant norovirus subtypes in Shenzhen was identified and corroborated, which was also a new discovery and highlight of our study.

Moreover, future research needs to expand the sample size, improve the collection of clinical data, and combine multiple analytical methods to more comprehensively explore the evolutionary mechanisms of norovirus and its potential impact on public health.

Overall, long-term monitoring studies in Shenzhen, we have identified the diverse characteristics of noroviruses in the Shenzhen region, investigated their evolutionary patterns, and explored mechanisms of mutation and recombination. Particularly, the norovirus in Shenzhen shares similarities with global epidemic characteristics in various aspects, while also exhibiting its own unique features. For the purpose of tracking and comprehending the epidemiology, evolution, and transmission of norovirus in South China and Shenzhen regions, this investigation is crucial. Furthermore, this research contributes genomic sequence of norovirus strains in Shenzhen, which have enriched the information available and will facilitate future investigations of the genomic characteristics of norovirus.

## 5 Conclusion

This research has conducted a thorough analysis of the distribution and genetic diversity of HuNoV in Shenzhen from 2016 to 2022 based on the sentinel surveillance network. The demographic features of norovirus infections in Shenzhen showed infect children under three and adults aged 20–40, with a higher prevalence in males. For spatial and temporal distribution, norovirus epidemic exhibited a seasonal pattern, with a greater propensity to become prevalent in highly inhabited and economically developed regions in Shenzhen. Phylogenetic analysis distinguished two genogroups of HuNoV, with GI strains generally existing in sporadic forms, while GII is frequently prevalent in several regions. In the GII group, multiple dominant genotypes originate from Japan and the United States. They exhibited similar or different evolutionary trends across geographical levels. Particularly, the GII.4_Sydney[P31] strain may exhibit a potential trend of periodic transmission at the level of Shenzhen. Based on the analysis of antigenic and protein stability, this hypothesis has been validated by mutational studies among GII.4_Sydney[P31]. Additionally, recombination analysis discovered the diversity of recombination events and identified a recombination-prone region, where breakpoints and fragment lengths exhibit a relatively consistent pattern. This genetic analysis of norovirus isolated in Shenzhen has expanded our understanding of the virus, contributing new sequence data to enrich database resources and providing valuable insights into the evolution and transmission of norovirus. Moreover, continuous monitoring and subsequent research are crucial to promote the development and implementation of vaccines, as well as to prevent or control potential disease outbreaks.

## Data Availability

The datasets presented in this study can be found in online repositories. The names of the repository/repositories and accession number(s) can be found in the article/**Supplementary Material**.

## References

[B1] Ahmed S. M. Hall A. J. Robinson A. E. Verhoef L. Premkumar P. Parashar U. D.. (2014). Global prevalence of norovirus in cases of gastroenteritis: a systematic review and meta-analysis. Lancet Infect. Dis. 14, 725–730. doi: 10.1016/S1473-3099(14)70767-4 24981041 PMC8006533

[B2] Bankevich A. Nurk S. Antipov D. Gurevich A. A. Dvorkin M. Kulikov A. S.. (2012). SPAdes: a new genome assembly algorithm and its applications to single-cell sequencing. J. Comput. Biol. 19, 455–477. doi: 10.1089/cmb.2012.0021 22506599 PMC3342519

[B3] Bolger A. M. Lohse M. Usadel B. (2014). Trimmomatic: a flexible trimmer for Illumina sequence data. Bioinf. (Oxford. England). 30, 2114–2120. doi: 10.1093/bioinformatics/btu170 PMC4103590 24695404

[B4] Bull R. A. Eden J. S. Rawlinson W. D. White P. A. (2010). Rapid evolution of pandemic noroviruses of the GII.4 lineage. PloS Pathog. 6, e1000831. doi: 10.1371/journal.ppat.1000831 20360972 PMC2847951

[B5] Bull R. A. Hansman G. S. Clancy L. E. Tanaka M. M. Rawlinson W. D. White P. A. (2005). Norovirus recombination in ORF1/ORF2 overlap. Emerging. Infect. Dis. 11, 1079–1085. doi: 10.3201/eid1107.041273 PMC3371806 16022784

[B6] Campillay-Véliz C. P. Carvajal J. J. Avellaneda A. M. Escobar D. Covián C. Kalergis A. M.. (2020). Human norovirus proteins: implications in the replicative cycle, pathogenesis, and the host immune response. Front. Immunol. 11, 961. doi: 10.3389/fimmu.2020.00961 32612600 PMC7308418

[B7] Chen L. N. Wang S. J. Wang S. M. Fu X. L. Zheng W. J. Hao Z. Y.. (2023). Molecular epidemiology analysis of symptomatic and asymptomatic norovirus infections in Chinese infants. Virol. J. 20, 60. doi: 10.1186/s12985-023-02024-z 37016444 PMC10074819

[B8] Chhabra P. de Graaf M. Parra G. I. Chan M. C. Green K. Martella V.. (2019). Updated classification of norovirus genogroups and genotypes. J. Gen. Virol. 100, 1393–1406. doi: 10.1099/jgv.0.001318 31483239 PMC7011714

[B9] Debbink K. Donaldson E. F. Lindesmith L. C. Baric R. S. (2012). Genetic mapping of a highly variable norovirus GII.4 blockade epitope: potential role in escape from human herd immunity. J. Virol. 86, 1214–1226. doi: 10.1128/JVI.06189-11 22090110 PMC3255819

[B10] Donaldson E. F. Lindesmith L. C. Lobue A. D. Baric R. S. (2010). Viral shape-shifting: norovirus evasion of the human immune system. Nat. Rev. Microbiol. 8, 231–241. doi: 10.1038/nrmicro2296 20125087 PMC7097584

[B11] Giammanco G. M. De Grazia S. Bonura F. Cappa V. Muli S. L. Pepe A.. (2017). Norovirus GII.17 as major epidemic strain in Italy, winter 2015-16. Emerging. Infect. Dis. 23, 1206–1208. doi: 10.3201/eid2307.161255 PMC5512478 28628440

[B12] He Y. Jin M. Chen K. Zhang H. Yang H. Zhuo F.. (2016). Gastroenteritis outbreaks associated with the emergence of the new GII.4 sydney norovirus variant during the epidemic of 2012/13 in shenzhen city, China. PloS One 11, e0165880. doi: 10.1371/journal.pone.0165880 27829005 PMC5102426

[B13] Hu X. He Y. Jin Y. Ma H. Xu B. Shao C.. (2022). Genetic diversity and persistent transmission of norovirus in Shenzhen, China, 2016–2018. J. Infect. 84, e89–e91. doi: 10.1016/j.jinf.2022.01.018 35041921

[B14] Humphrey W. Dalke A. Schulten K. (1996). VMD: visual molecular dynamics. J. Mol. Graphics 14, 33–38, 27-38. doi: 10.1016/0263-7855(96)00018-5 8744570

[B15] Kapikian A. Z. Wyatt R. G. Dolin R. Thornhill T. S. Kalica A. R. Chanock R. M. (1972). Visualization by immune electron microscopy of a 27-nm particle associated with acute infectious nonbacterial gastroenteritis. J. Virol. 10, 1075–1081. doi: 10.1128/jvi.10.5.1075-1081.1972 4117963 PMC356579

[B16] Katoh K. Misawa K. Kuma K. and Miyata T. (2002). MAFFT: a novel method for rapid multiple sequence alignment based on fast Fourier transform. Nucleic Acids Res. 30, 3059–3066. doi: 10.1093/nar/gkf436 12136088 PMC135756

[B17] Kearse M. Moir R. Wilson A. Stones-Havas S. Cheung M. Sturrock S.. (2012). Geneious Basic: an integrated and extendable desktop software platform for the organization and analysis of sequence data. Bioinf. (Oxford. England). 28, 1647–1649. doi: 10.1093/bioinformatics/bts199 PMC3371832 22543367

[B18] Kitajima M. Oka T. Haramoto E. Takeda N. Katayama K. Katayama H. (2010). Seasonal distribution and genetic diversity of genogroups I, II, and IV noroviruses in the Tamagawa River, Japan. Environ. Sci. Technol. 44, 7116–7122. doi: 10.1021/es100346a 20715862

[B19] Li D. Luo R. Liu C. M. Leung C. M. Ting H. F. Sadakane K.. (2016). MEGAHIT v1.0: A fast and scalable metagenome assembler driven by advanced methodologies and community practices. Methods (San Diego Calif). 102, 3–11. doi: 10.1016/j.ymeth.2016.02.020 27012178

[B20] Lindesmith L. C. Costantini V. Swanstrom J. Debbink K. Donaldson E. F. Vinjé J.. (2013). Emergence of a norovirus GII.4 strain correlates with changes in evolving blockade epitopes. J. Virol. 87, 2803–2813. doi: 10.1128/JVI.03106-12 23269783 PMC3571402

[B21] Liu L. Oza S. Hogan D. Perin J. Rudan I. Lawn J. E.. (2015). Global, regional, and national causes of child mortality in 2000-13, with projections to inform post-2015 priorities: an updated systematic analysis. Lancet (London England). 385, 430–440. doi: 10.1016/S0140-6736(14)61698-6 25280870

[B22] Lopman B. A. Steele D. Kirkwood C. D. Parashar U. D. (2016). The vast and varied global burden of norovirus: prospects for prevention and control. PloS Med. 13, e1001999. doi: 10.1371/journal.pmed.1001999 27115709 PMC4846155

[B23] Lun J. H. Hewitt J. Yan G. J. H. Enosi Tuipulotu D. Rawlinson W. D. White P. A. (2018). Recombinant GII.P16/GII.4 sydney 2012 was the dominant norovirus identified in Australia and New Zealand in 2017. Viruses 10, 548. doi: 10.3390/v10100548 30304780 PMC6213408

[B24] Martin D. P. Murrell B. Golden M. Khoosal A. Muhire B. (2015). RDP4: Detection and analysis of recombination patterns in virus genomes. Virus Evol. 1, vev003. doi: 10.1093/ve/vev003 27774277 PMC5014473

[B25] Nguyen L. T. Schmidt H. A. von Haeseler A. Minh B. Q. (2015). IQ-TREE: a fast and effective stochastic algorithm for estimating maximum-likelihood phylogenies. Mol. Biol. Evol. 32, 268–274. doi: 10.1093/molbev/msu300 25371430 PMC4271533

[B26] Ouedraogo N. Ngangas S. M. Bonkoungou I. J. Tiendrebeogo A. B. Traore K. A. Sanou I.. (2017). Temporal distribution of gastroenteritis viruses in Ouagadougou, Burkina Faso: seasonality of rotavirus. BMC Public Health 17, 274. doi: 10.1186/s12889-017-4161-7 28327111 PMC5359802

[B27] Patel M. M. Hall A. J. Vinjé J. Parashar U. D. (2009). Noroviruses: a comprehensive review. J. Clin. Virol. 44, 1–8. doi: 10.1016/j.jcv.2008.10.009 19084472

[B28] Pazdiora P. Jelínková H. Bartoníková N. Gartnerová E. Kudová J. Vidličková I.. (2022). Norovirus infections in the Czech Republic in 2008-2020. Epidemiol. Mikrobiol. Imunol. 71, 78–85. 35940861

[B29] Pérez-Losada M. Arenas M. Galán J. C. Palero F. González-Candelas F. (2015). Recombination in viruses: mechanisms, methods of study, and evolutionary consequences. Infect. Genet. Evol. 30, 296–307. doi: 10.1016/j.meegid.2014.12.022 25541518 PMC7106159

[B30] Pires S. M. Fischer-Walker C. L. Lanata C. F. Devleesschauwer B. Hall A. J. Kirk M. D.. (2015). Aetiology-specific estimates of the global and regional incidence and mortality of diarrhoeal diseases commonly transmitted through food. PloS One 10, e0142927. doi: 10.1371/journal.pone.0142927 26632843 PMC4668836

[B31] Rydell G. E. Kindberg E. Larson G. Svensson L. (2011). Susceptibility to winter vomiting disease: a sweet matter. Rev. Med. Virol. 21, 370–382. doi: 10.1002/rmv.v21.6 22025362

[B32] Shah M. P. Hall A. J. (2018). Norovirus illnesses in children and adolescents. Infect. Dis. Clinics North America 32, 103–118. doi: 10.1016/j.idc.2017.11.004 PMC6814392 29406972

[B33] Shanker S. Choi J. M. Sankaran B. Atmar R. L. Estes M. K. Prasad B. V. (2011). Structural analysis of histo-blood group antigen binding specificity in a norovirus GII.4 epidemic variant: implications for epochal evolution. J. Virol. 85, 8635–8645. doi: 10.1128/JVI.00848-11 21715503 PMC3165782

[B34] Suchard M. A. Lemey P. Baele G. Ayres D. L. Drummond A. J. Rambaut A. (2018). Bayesian phylogenetic and phylodynamic data integration using BEAST 1.10. Virus Evol. 4, vey016. doi: 10.1093/ve/vey016 29942656 PMC6007674

[B35] Tohma K. Lepore C. J. Martinez M. Degiuseppe J. I. Khamrin P. Saito M.. (2021). Genome-wide analyses of human noroviruses provide insights on evolutionary dynamics and evidence of coexisting viral populations evolving under recombination constraints. PloS Pathog. 17, e1009744. doi: 10.1371/journal.ppat.1009744 34255807 PMC8318288

[B36] Vinjé J. (2015). Advances in laboratory methods for detection and typing of norovirus. J. Clin. Microbiol. 53, 373–381. doi: 10.1128/JCM.01535-14 24989606 PMC4298492

[B37] White P. A. (2014). Evolution of norovirus. Clin. Microbiol. Infect. 20, 741–745. doi: 10.1111/1469-0691.12746 24980204

[B38] Zhang G. Wang J. Liu J. Zheng L. Wang W. Huo Y.. (2019). The surface-exposed loop region of norovirus GII.3 VP1 plays an essential role in binding histo-blood group antigens. Arch. Virol. 164, 1629–1638. doi: 10.1007/s00705-019-04256-3 30968211

[B39] Zheng G. L. Zhu Z. X. Cui J. L. Yu J. M. (2022). Evolutionary analyses of emerging GII.2P16. and GII.4 Sydney [P16] noroviruses. Virus Evol. 8, veac030. doi: 10.1093/ve/veac030 35450165 PMC9019527

[B40] Zhou H. Wang S. von Seidlein L. Wang X. (2020). The epidemiology of norovirus gastroenteritis in China: disease burden and distribution of genotypes. Front. Med. 14, 1–7. doi: 10.1007/s11684-019-0733-5 31823287 PMC8320309

[B41] Zhu X. He Y. Wei X. Kong X. Zhang Q. Li J.. (2021). Molecular epidemiological characteristics of gastroenteritis outbreaks caused by norovirus GII.4 sydney P31. Strains - China, October 2016-December 2020. China CDC. Wkly. 3, 1127–1132. doi: 10.46234/ccdcw2021.276 35036035 PMC8742140

