# Supplementary material for: Genetic diversity of norovirus in Shenzhen Based on continuous surveillance from 2016 to 2022
Source: Front Cell Infect Microbiol. 2025 Jun 18;15:1593610. doi: 10.3389/fcimb.2025.1593610 (PMC12213568; doi:10.3389/fcimb.2025.1593610)
Supplement: Supplementary file 1 [file DataSheet1.zip › supplementary material/Table S2.docx]

Table S2. Details of recombination events detected by at least five methods with RDP based on alignment of the norovirus whole genome

| **Event** | **Recombinant(R) and Parents (P1, P2)** | **No.of methods** | **P Value range** | **breakpoints position in Recombinant Sequence** |
| --- | --- | --- | --- | --- |
| 1 | C_AA085002.1_CHN_2021_GII.4[P16]  S16-411_ CHN_2016_GII.2[P16]  MH671553_ CHN_2016_GII.2[P2] | 5 | 2.98E-217–1.54E-09 | Begin: 4274  End: 6720 |
| 2 | LC209435_JP_2004_GII.2[P12] ^a^  KC662537_KR_GII.13[P12]  LC209463_JP_2008_GII.2[P2] | 5 | 1.67E-136–  1.21E-09 | Begin: 5088  End: 7526 |
| 3 | KF944111_RUS_2011_GII.3[P16] ^a^  LC209451_JP_2011_GII.2[P16]  MH218655_NK_2015_GII.2[P2] | 5 | 3.50E-205–  1.59E-09 | Begin: 5080  End: 7492 |
| 4 | LC597126_ID_2016_ GII.13[P16] ^a^  LC209451_JP_2011_GII.2[P16]  MH218655_UK_2015_GII.2[P2] | 5 | 2.28E-202–  1.06E-09 | Begin: 5101  End: - |
| 5 | KF306213_CHN_2013_GII.3[P12] ^a^  AB220921_JP_2005_GII.4[P12]  MH218573_UK_2014_GII.3[P21] | 5 | 2.87E-109–  1.06E-09 | Begin: 5089  End:7541 |
| 6 | LC597120_ID_2015_GII.6[P7] ^a^  S18-435_CHN_2018_GII.2[P16]  MK753034_USA_2017_GII.1[P16] | 5 | 8.22E-168–  1.59E-09 | Begin: 5074  End:7465 |
| 7 | KJ196294_JP_2000_GII.12[P12] ^a^  AB220921_JP_2005_GII.4[P12]  C_AA085041.1_CHN_2022_GII.12[P16] | 5 | 1.57E-126–  1.59E-09 | Begin: 5078  End: 7512 |
| 8 | S18-207_CHN_2018_GII.3[P12] ^a^  AB220921_JP_2005_GII.4[P12]  AB365435_USA_2004_GII.3[P21] | 5 | 8.86E-122–  1.06E-09 | Begin: 5093  End: 7525 |
| 9 | C_AA085041.1_CHN_2022_GII.12[P16] a  C_AA084944.1_CHN_2020_GII.2[P16]  HQ664990_USA_2010_GII.12[P33] | 5 | 7.86E-126–  1.21E-09 | Begin: 4906  End: 7348 |
| 10 | AB365435_USA_2004_GII.3[P21] a  KX079488_KR_2015_GII.21[P21]  C_AA084989.1_CHN_2021_GII.3[P25] | 5 | 3.55E-85–  1.89E-09 | Begin: 5110  End: 7540 |
| 11 | C_AA084887.1_CHN_2021_GII.4[P31] a  C_AA084924.1_CHN_2020_GII.17[P31]  C_AA084966.1_CHN_2020_GII.17[P17] | 5 | 1.06E-27–  1.19E-03 | Begin: 4980  End: - |
| 12 | LC342058_Japan_1998_GI.6 ^a^  S17-134_CHN_2017_GI.1[P1]  S18-077_CHN_2018_GI.6[P11] | 5 | 9.66E-55–  4.37E-04 | Begin: 5350  End: - |
| 13 | C_AA085029.1_CHN_2022_GI.2[P2]  S17-134_CHN_2017_GI.1[P1]  C_AA085033.1_CHN_2022_GI.6[P11] | 5 | 2.39E-45–  2.09E-02 | Begin: 2868  End: 3645 |
| 14 | C_AA084988.1_CHN_2021_GII.4[P16]  C_AA084981.1_CHN_2021_GII.4[P16]  C_AA085008.1_CHN_2021_GII.4[P31] | 5 | 7.51E-70–  1.59E-09 | Begin: 1750  End: 2209 |
| 15 | MT372463_USA_2016_GI.3[P3] ^a^  S18-236_CHN_2018_GI.3[P13]  S18-062_CHN_2018_GI.3[P13] | 5 | 3.32E-27–  8.77E-07 | Begin: 6098  End: - |
| 16 | C_AA085004.1_CHN_2021_GII.4[P16]  C_AA084987.1_CHN_2021_GII.4[P16]  C_AA085014.1_CHN_2021_GII.4[P31] | 5 | 7.93E-56–  1.59E-09 | Begin: 2207  End: 2480 |
| 17 | C_AA085029.1_CHN_2022_GI.2[P2]  S17-134_CHN_2017_GI.1[P1]  C_AA085033.1_CHN_2022_GI.6[P11] | 5 | 8.59E-26–  1.14E-02 | Begin: 4463  End: 4687 |
| 18 | C_AA085029.1_CHN_2022_GI.2[P2]  S17-134_CHN_2017_GI.1[P1]  MT372464_USA_2017_GI.2[P2] | 5 | 3.97E-35–  5.65E-05 | Begin: 6793  End: 6993 |
| 19 | MH671553_CHN_2016_GII.2[P2] ^a^  JN400618_CHN_2009_GII.4[P4]  S17-227_CHN_2017_GII.2[P16] | 5 | 3.98E-75–  7.75E-06 | Begin: 5080  End: 7527 |
| 20 | AY134748_USA_2002_GII.2[P30]  JN400618_CHN_2009_GII.4[P4]  S17-227_CHN_2017_GII.2[P16] | 5 | 1.79E-60–  5.30E-10 | Begin: 5078  End: 7528 |
| 21 | MF405169_USA_1971_GII.2[Pf]  JN400618_CHN_2009_GII.4[P4]  S17-227_CHN_2017_GII.2[P16] | 5 | 5.84E-38–  2.56E-07 | Begin: 5080  End: 7527 |

^a^ An apparent recombination signal possibly caused by an evolutionary event other than recombination.

^b^ The actual breakpoint position (undetermined) was most likely either overprinted by a subsequent recombination event or off the edges of the analyzed sequence fragments.
