# Supplementary material for: Genetic diversity of norovirus in Shenzhen Based on continuous surveillance from 2016 to 2022
Source: Front Cell Infect Microbiol. 2025 Jun 18;15:1593610. doi: 10.3389/fcimb.2025.1593610 (PMC12213568; doi:10.3389/fcimb.2025.1593610)
Supplement: Supplementary file 2 [file DataSheet2.zip › Supplementary file 2/Figure S2.docx]

Figure S2. Maximum Clade Credibility (MCC) tree of the human norovirus RdRp gene. The branches of the tree are colored according to the geographical location, with purple and green lines representing GII and GI genotypes, respectively. Shenzhen strains are marked with red pentagrams, and the dominant genotypes in this study are highlighted with boxes. The rectangular boxes on the nodes represent the credibility intervals, and the numbers indicate the posterior probability values.
